# Impact of Preoperative Anaemia on Blood Transfusion and Clinical Outcomes in Total Knee Arthroplasty: A Retrospective Observational Study

**DOI:** 10.4274/TJAR.2026.252329

**Published:** 2026-04-15

**Authors:** Gülencan Yumuşak Ergin, Ayşe Tıraş Çetin, Asiye Ceylan, Sümeyye Önal Altınkaya, Eyyüp Çetin

**Affiliations:** 1 Aksaray University Training and Research Hospital Department of Anesthesiology and Reanimation, Aksaray, Türkiye

**Keywords:** Anaemia, blood transfusion, perioperative care

## Abstract

**Objective:**

Preoperative anemia is a common, yet inadequately managed condition in patients undergoing total knee arthroplasty (TKA) and is associated with an increased need for perioperative blood transfusions. However, variability in physicians’ transfusion practices remains understudied. This study investigated the influence of preoperative anaemia on transfusion rates and clinical outcomes and examined inter-physician variability in transfusion procedures.

**Methods:**

This study included 265 patients who underwent TKA. Preoperative anaemia was defined as haemoglobin <13 g dL^-1^. Demographic characteristics, perioperative variables, laboratory parameters, transfusion data, and postoperative outcomes were recorded. Transfusion rates, complications, and lengths of hospital stay were compared between anemic and non-anemic groups. Inter-physician variability in transfusion decisions was also analysed.

**Results:**

Preoperative anaemia was present in 43% of individuals. Transfusion rates were significantly higher in patients with anaemia (69.3% vs. 54.3%, *P*=0.013). When postoperative outcomes were analysed according to anaemia and transfusion status, anaemia was not independently associated with postoperative complications (*P*=0.072). Perioperative blood transfusion was associated with significantly higher complication rates (31.7% vs. 15.4%, *P*=0.003) and a prolonged hospital stay (*P* < 0.001). Receiver operating characteristic analysis showed modest discrimination for predicting transfusion (area under the receiver operating characteristic curve =0.61; cut-off =13.15 g dL^-1^). Significant inter-physician variability was observed, independent of anaemia status (*P* < 0.05).

**Conclusion:**

Preoperative anemia is common among TKA patients and has been associated with higher transfusion rates. Transfusion was associated with adverse clinical outcomes, including prolonged hospitalisation and higher complication rates. The substantial physician-related variability observed in transfusion practices underscores the need for standardised, evidence-based perioperative transfusion protocols.

**Main Points**

• Anaemia is common among patients undergoing total knee arthroplasty and contributes significantly to the need for blood transfusions.

• Blood transfusions are linked to greater postoperative morbidity and longer hospital stays.

• Substantial physician-related variability exists in transfusion practices, underscoring the need for standardised, evidence-based perioperative transfusion protocols and comprehensive patient blood management strategies.

## Introduction

Total knee arthroplasty (TKA) is commonly used to relieve pain and restore function in individuals with advanced knee osteoarthritis. The lifetime prevalence of symptomatic knee arthritis is estimated to be as high as 44.7%.^1^ Population ageing, together with an increasing number of primary procedures, has resulted in a growing demand for revision TKA. This trend is driven by the success of primary TKAs, patients’ desire to maintain active lifestyles, and the expansion of surgical indications to include younger individuals.^2^ Despite its well-established benefits and excellent long-term outcomes, including implant survival exceeding 90%, TKA remains associated with substantial perioperative risks, including significant blood loss, cardiovascular complications, and other postoperative adverse events.^3^

A primary objective of perioperative care is to optimise patient outcomes by reducing morbidity and mortality while facilitating early mobilisation and shorter hospital stays.^4^ Among these optimisation strategies, the management of preoperative anaemia has gained increasing attention. It is most commonly associated with iron deficiency.^5^ The frequency of anaemia rises with advancing age and reaches particularly high levels in individuals over 65 years.^6^ Preoperative anemia is commonly seen in patients undergoing major orthopaedic procedures, such as TKA,^7, 8, 9^ and is consistently linked to elevated postoperative morbidity, mortality, and a greater likelihood of requiring allogeneic blood transfusions.^10, 11, 12^ Timely recognition and appropriate management of preoperative anaemia are critical for optimising surgical readiness, reducing transfusion requirements, and improving clinical outcomes. In this context, patient blood management (PBM) provides a structured, evidence-based strategy designed to correct anaemia and minimise blood loss. PBM also aims to optimise the utilisation of blood products and improve overall perioperative outcomes. The implementation of PBM has been shown to enhance patient safety, reduce transfusion-related complications, and improve cost-effectiveness.^13^ However, in real-world clinical practice, preoperative anaemia is often overlooked, resulting in potentially avoidable transfusions and increased exposure to associated risks. Additionally, transfusion decision-making varies substantially among physicians and is often guided by individual clinical judgement rather than standardised criteria.^14^ This inconsistency represents an important quality-of-care concern.

In major orthopaedic surgery, perioperative blood loss and transfusion remain important clinical concerns, prompting the adoption of evidence-based blood management strategies. Prophylactic use of antifibrinolytic agents, particularly tranexamic acid administered orally, intravenously, and/or topically, has been shown to effectively reduce perioperative blood loss and transfusion requirements in patients undergoing high-bleeding-risk orthopaedic procedures. In contrast, routine use of intraoperative tourniquets or surgical drains has not consistently demonstrated a reduction in overall perioperative blood loss or transfusion rates in TKA.^15^

Current recommendations further emphasize restrictive transfusion approaches and goal-directed decision-making based on haemoglobin concentrations and physiological indicators rather than fixed numerical thresholds. Restrictive strategies generally support haemoglobin targets of approximately 7-8 g dL^-1^ in clinically stable patients, with higher targets reserved for those with ongoing bleeding or limited cardiopulmonary reserve.^15, 16^

In orthopaedic patients, particularly those undergoing major joint arthroplasty, early identification of perioperative anaemia, postoperative haemoglobin monitoring, and timely intravenous iron supplementation when indicated are considered essential components of PBM.^15^ Despite the availability of evidence-based perioperative blood management strategies, substantial variability in transfusion thresholds and continued reliance on traditional haemoglobin triggers persist in orthopaedic practice, providing a rationale for further evaluation of perioperative anaemia and transfusion patterns in total joint arthroplasty.

This study aims to assess the impact of untreated preoperative anaemia on perioperative transfusion rates and clinical outcomes in individuals undergoing primary TKA. In addition, physician-related variability in transfusion decisions is evaluated to highlight the influence of individual clinical decision-making on perioperative blood management.

## Methods

### Study Design and Setting

This research was carried out as a single-centre retrospective observational study. Ethical approval has been obtained from the Aksaray University Health Sciences Scientific Research Ethics Committee (approval no.: SAGATİK 2025-001, date: 30.01.2025). The study population consisted of patients who underwent TKA between January and December 2024 at Aksaray University Training and Research Hospital.

During the study period, no standardized institutional transfusion protocol was in place. Transfusion decisions were made by the attending surgeon or anaesthesiologist based on individual clinical judgment, including perioperative haemoglobin levels and overall patient condition.

### Patient Selection

**Figure 1:**
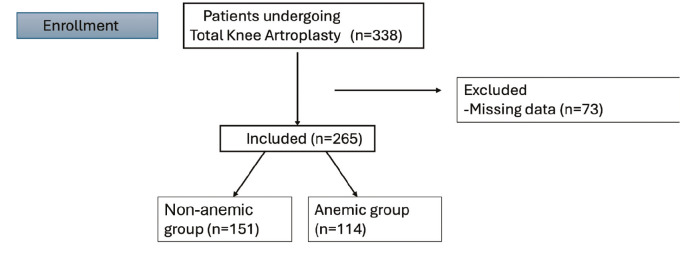
Patient flow diagram of the study population.

Adult patients (≥18 years) who underwent primary or revision TKA were eligible for inclusion. Patients with incomplete or missing medical records were excluded. Figure 1 illustrates the patient selection process.

### Data Collection

Patient information was retrieved from electronic medical records and institutional patient files. Demographic variables, including age, sex, and body mass index (BMI), as well as the American Society of Anaesthesiologists’ physical status and comorbidities, were recorded. Perioperative variables included laterality (unilateral or bilateral), surgical duration, anaesthesia modality (regional or general), and laboratory parameters. Preoperative and postoperative haemoglobin and creatinine levels were measured. Transfusion data included timing (intraoperative, postoperative, or both) and the number of red blood cell units administered. Postoperative outcomes included complications, intensive care unit admission, length of hospital stay, and in-hospital mortality. The operating surgeon and the anaesthesiologist for each case were documented.

Postoperative events were graded according to the Clavien-Dindo system, and complications at grade 2 or higher were taken as clinically relevant.^17^ Preoperative anaemia was defined as a haemoglobin concentration <13 g dL^-1^ in male and female patients.^18, 19^

### Statistical Analysis

Categorical data were expressed as numbers and percentages. Continuous variables were summarised as mean ± standard deviation or median (range), depending on their distribution. Group comparisons were made using chi-square test or Fisher’s exact test for categorical variables, and t-test or Mann-Whitney U test for continuous variables. When more than two non-normally distributed groups were analysed, the Kruskal-Wallis test was used.

Baseline haemoglobin as a predictor of transfusion was evaluated with receiver operating characteristic (ROC) analysis, and the area under the curve (AUC) and optimal cut-off (Youden index) were calculated. Diagnostic accuracy at this cut-off was also reported. The relationship between haemoglobin level and the number of transfused units was assessed using Spearman’s rank correlation coefficient. In addition, multivariable logistic regression analysis was performed to identify factors independently associated with perioperative blood transfusion. Model fit was assessed using the Hosmer-Lemeshow goodness-of-fit test, and the proportion of variance explained by the model was evaluated using Cox & Snell and Nagelkerke R² statistics. A *P* value of <0.05 was considered statistically significant. Analyses were performed with SPSS version 29.0 (IBM Corp., Armonk, NY, USA).

## Results

A total of 265 patients were analyzed. The median age was 67 years (range: 45-89), and the median BMI was 31.3 kg m^2-1^ (range: 22.0-47.8). Most patients were female (89.4%). Hypertension was the most common comorbidity (54.7%), followed by diabetes mellitus (42.3%), coronary artery disease (22.3%), pulmonary disease (18.5%), and chronic kidney disease (3.0%). Regional anaesthesia was the predominant anaesthetic technique (90.6%). Most patients (90.6%) underwent primary surgery, whereas 9.4% underwent revision procedures. Table 1 summarises the baseline demographic and clinical characteristics.

Preoperative anaemia was present in 114 patients (43.0%). The Median baseline haemoglobin level for the entire cohort was 13.2 g dL^-1^ (range, 9.1-18.3). Baseline demographic profiles were similar in patients with and without anaemia (Table 2). Haemoglobin values differed significantly between the groups at all measured time points (Table 3). Median preoperative haemoglobin was 13.8 g dL^-1^ (range: 13.0-18.3) in the non-anaemic group and 12.2 g dL^-1^ (range: 9.1-12.9) in the anaemic group (*P* < 0.001). Mean postoperative haemoglobin was also significantly lower in patients with anaemia (10.5±1.2 g dL^-1^ vs. 12.2±1.2 g dL^-1^, *P* < 0.001). The nadir haemoglobin level during hospitalisation was lower in the anaemic group [9.1 g dL^-1^ (6.7-12.5) vs. 9.9 g dL^-1^ (5.7-13.6), *P* < 0.001]. At discharge, mean haemoglobin concentration remained significantly lower among patients with anaemia (10.2±0.9 g dL^-1^ vs. 10.6±1.2 g dL^-1^; *P*=0.002).

At least one unit of red blood cell transfusion was administered to 69.3% of patients with anaemia and 54.3% of patients without anaemia (*P*=0.013). Among patients who received transfusions, no significant difference was found in transfusion timing between groups (*P*=0.642). Intraoperative-only transfusions were performed in 13.4% of patients without anaemia and in 8.9% of patients with anaemia, whereas postoperative-only transfusions were the most common in both groups (75.6% and 78.5%, respectively). Combined intraoperative and postoperative transfusions occurred in 11.0% and 12.7% of patients in the non-anaemic and anaemic groups, respectively (Table 4). A statistically significant inverse relationship was observed between baseline hemoglobin levels and the number of blood units transfused. (r =-0.248, *P *< 0.001).

In the adjusted analysis, multivariable logistic regression was performed to identify factors independently associated with perioperative blood transfusion (Table 5). Increasing age was independently associated with a higher likelihood of transfusion [odds ratio (OR): 1.06, 95% confidence interval (CI): 1.02-1.10; *P*=0.002]. Bilateral surgery emerged as the strongest independent predictor of transfusion requirement (OR: 5.98, 95% CI: 3.13-11.43; *P *< 0.001). Coronary artery disease was associated with increased odds of transfusion (OR: 2.61, 95% CI: 1.18-5.77, *P*=0.018), whereas other comorbidities, revision surgery, and anaesthesia type were not independently associated with transfusion.

Clinical outcomes were assessed based on anaemia status and transfusion exposure. Complication rates were initially analysed using the standard Clavien-Dindo classification (CDC). A secondary analysis was subsequently performed, excluding blood transfusion from the composite outcome to evaluate postoperative complications other than transfusion.

According to the standard composite outcome, the overall rate of CDC grade ≥2 complications was numerically higher in the anaemic group (66.7% vs. 54.9%), although this difference did not reach statistical significance (*P*=0.072). After excluding blood transfusion from the composite outcome, complication rates were nearly identical between groups (25.4% vs. 25.2%; *P*=0.960). Acute kidney injury occurred more frequently in patients with anaemia than in those without (2.6% vs. 0%), but this difference did not reach statistical significance (*P*=0.094). Intensive care unit admission rates were similar between groups (6.0% vs. 8.8%; *P*=0.380). The median length of hospital stay did not change substantially [7 days (range: 0-20) vs. 7 days (range: 2-60); *P*=0.183]. Additionally, the proportion of patients with prolonged hospitalization (≥10 days) was comparable (9.9% vs. 14.9%; *P*=0.218).

When clinical outcomes were analysed according to transfusion status, transfused patients exhibited significantly higher rates of CDC grade ≥2 complications, both when blood transfusion was included in the Clavien-Dindo composite outcome (96.5% vs. 17.4%, *P* < 0.001) and when it was excluded (31.7% vs. 15.4%, *P*=0.003). Although acute kidney injury occurred more frequently among transfused patients (10.6% vs. 4.8%), this difference was not statistically significant (*P*=0.152). Intensive care unit admission rates were comparable between the groups (8.1% vs. 5.8; *P*=0.478). However, transfused patients had a significantly longer median hospital stay [7 days (range: 3-60) vs. 6 days (range: 0-13); *P* < 0.001] and a greater proportion experienced prolonged hospitalisation (≥10 days: 17.4% vs. 3.8%; *P* < 0.001).

No significant differences were observed in complication rates between unilateral and bilateral procedures (*P*=0.222). The length of hospital stay did not differ significantly (*P*=0.057), although the *P* value was close to the threshold for significance, suggesting a possible trend toward longer hospitalisation after bilateral surgery.

**Figure 2:**
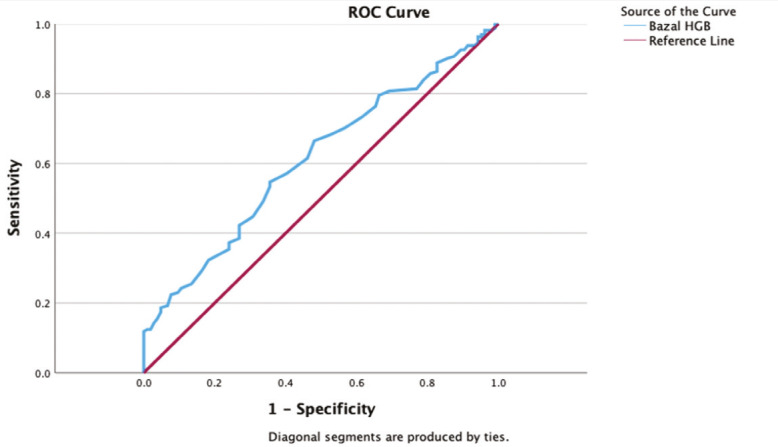
ROC curve analysis of haemoglobin thresholds for predicting perioperative transfusion requirements. ROC, receiver operating characteristic; HGB, hemoglobin.

The ability of baseline haemoglobin to predict perioperative blood transfusion was evaluated using ROC analysis (Figure 2). The AUC was 0.61 (95% CI: 0.54-0.68, *P*=0.002), indicating modest discriminative ability. The optimal cut-off value was 13.15 g dL^-1^, yielding a sensitivity of 55%, specificity of 64%, positive predictive value of 70%, negative predictive value of 48%, and an overall accuracy of 58% (Table 6).

**Figure 3:**
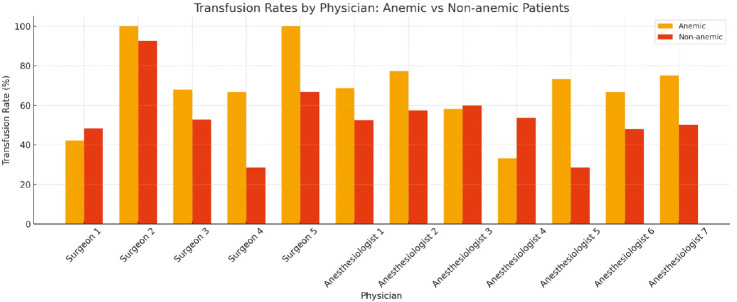
Variation in transfusion rates among surgeons and anaesthesiologists, stratified by anaemia status.

Only surgeons and anaesthesiologists who managed at least 10 patients were included in the analysis of physician-related transfusion practices. Marked variability in transfusion rates was observed across physicians. Among surgeons, the proportion of patients with anaemia receiving transfusions ranged from 42.1% to 100%, and from 28.6% to 92.6% among patients without anaemia. Similarly, anaesthesiologists displayed wide variation in transfusion rates ranging from 18.8% to 100% in patients with anaemia and from 28.6% to 75.0% in patients without anaemia (Figure 3). Statistical testing showed significant variability in transfusion decisions among physicians in both the anaemic and non-anaemic groups (*P* < 0.05).

## Discussion

This retrospective observational study investigated the impact of preoperative anaemia on transfusion requirements and clinical outcomes in patients undergoing TKA. The principal findings were that preoperative anaemia was common and significantly associated with an increased likelihood of perioperative blood transfusion, whereas transfusion itself was linked to worse postoperative outcomes. In addition, substantial physician-related variability in transfusion practices was observed, highlighting the need for greater standardisation in perioperative blood management. Taken together, these findings suggest that transfusion exposure, rather than preoperative anaemia itself, is more closely associated with adverse postoperative outcomes.

In this study, preoperative anaemia was defined as haemoglobin <13 g dL^-1^ in both sexes, adopting a uniform cut-off, consistent with contemporary perioperative anaemia definitions and recommendations that favour sex-independent criteria to enable standardised risk stratification, despite the widespread use of sex-specific thresholds.^18, 19^ Using this definition, 43% of patients were classified as anaemic, reinforcing the substantial burden of this modifiable risk factor in patients undergoing TKA. Consistent with previous studies, anaemia emerged as a major determinant of transfusion in our cohort.^10, 20, 21, 22^

Recent evidence indicates a progressive increase in the predictive value of anaemia for transfusion risk.^10^ Supporting this trend, a recent temporal analysis revealed that the OR for anaemia as a predictor of transfusion increased markedly over time, from 3.^1^ (95% CI: 2.1-4.6) in 2010 to 14.0 (95% CI: 8.9-24) in 2021, underscoring its growing importance in perioperative risk assessment.^10^ In our cohort, baseline haemoglobin demonstrated only modest ability to predict transfusion (AUC=0.61), yet the derived threshold (13.15 g dL^-1^) suggests that patients within the low-normal range may warrant closer attention. This finding aligns with emerging evidence indicating that lower preoperative haemoglobin is associated with impaired recovery and a higher risk of complications, even within contemporary perioperative care pathways.^23, 24^

Although previous studies have found a direct link between preoperative anaemia and postoperative morbidity^22, 23, 25^ this investigation found no statistically significant association between anaemia and overall complications. In contrast, transfused patients experienced significantly higher complication rates, suggesting that adverse outcomes may be more closely related to transfusion exposure than to anaemia alone.

Our findings are consistent with previous reports demonstrating associations with transfusion requirement, intensive care unit admission, prolonged hospitalisation, and major postoperative complications. Consistent with this, prior studies have reported an increased risk of adverse outcomes, including surgical site infection, sepsis, and pulmonary embolism, among transfused patients.^26, 27^ In our cohort, transfusion was similarly associated with a prolonged hospital stay, which is known to contribute to both clinical and economic burdens.^28^ Prolonged hospitalisation has also been linked to secondary complications, including delirium, particularly in older adults.^29^ However, postoperative cognitive outcomes could not be systematically evaluated in the present study due to limitations inherent in the retrospective design.

Our findings are consistent with the Turkish National Perioperative Transfusion Study (TULIP), a large, multicentre, observational study, nationwide. The TULIP study demonstrated substantial variability in perioperative transfusion practices among centres and clinicians and identified preoperative anaemia as a key determinant of transfusion requirements rather than as an independent predictor of adverse clinical outcomes. In addition, the wide variation in transfusion rates reported in the TULIP study was largely attributed to differences in transfusion thresholds and individual clinical decision-making.^14^ Taken together, these findings suggest that transfusion-related complications may reflect underlying patient characteristics and perioperative management strategies rather than preoperative anaemia itself. This observation further underscores the importance of evidence-based, standardized PBM approaches and closer collaboration between surgical and anaesthesia teams.

Although preoperative anaemia significantly increased the likelihood of perioperative blood transfusion, transfusion exposure was not confined to anaemic patients in our cohort. A substantial proportion of non-anaemic patients required transfusion, suggesting that transfusion decisions were influenced by perioperative factors beyond baseline haemoglobin level, such as intraoperative blood loss, bilateral procedures, and physician-dependent transfusion thresholds.

In the adjusted analysis, perioperative blood transfusion remained strongly influenced by patient- and procedure-related factors. Increasing age and bilateral surgery were identified as independent predictors of the need for transfusion, while coronary artery disease was additionally associated with an increased risk of transfusion. Importantly, most other comorbidities and the type of anaesthesia were not independently associated with transfusion after adjustment, suggesting that transfusion decisions are primarily driven by procedural complexity and patient vulnerability rather than the anaesthetic technique. Previous studies have similarly shown that age is an independent predictor of perioperative transfusion, with older patients exhibiting higher odds of transfusion despite adjustment for hemoglobin level and comorbidities.^30^

Similarly, surgical duration was significantly associated with transfusion, which may be explained by the longer operative time required for bilateral procedures. The risk-benefit profile of bilateral versus unilateral knee arthroplasty remains a topic of debate in the literature.^31, 32^ At our hospital, bilateral arthroplasty is often preferred for patients with multiple complaints or limited access to healthcare; however, no consensus exists among surgeons regarding the superiority of one technique over the other.

In our cohort, unilateral and bilateral procedures had comparable rates of postoperative complications, excluding transfusion, and similar lengths of hospital stay, suggesting that the increased transfusion requirements associated with bilateral procedures does not necessarily translate into a higher burden of postoperative morbidity. The lack of statistical significance in hospitalisation duration may reflect heterogeneity within patient subgroups.

When transfused patients were analyzed separately, transfusion decisions were made in both anaemic and non-anaemic groups, reinforcing that transfusion-related outcomes cannot be attributed solely to baseline anaemia status. In our cohort, substantial variability was observed in transfusion decisions among surgeons and anaesthesiologists. The wide variation among physicians, even after adjusting for anaemia status, suggests that transfusion practices may be influenced not only by clinical indications but also by individual decision-making styles. Such heterogeneity warrants attention, as it directly affects the consistency and quality of patient care and highlights the need for institutional, evidence-based transfusion frameworks. Intraoperative transfusion decisions are inherently multifactorial, shaped by physiological changes induced by anaesthesia, the surgical procedure, and acute intraoperative conditions, rather than by blood loss alone.^33^

Our findings highlight the relevance of systematic preoperative anaemia screening and transfusion practices in patients undergoing TKA. In the context of the observed association between transfusion exposure and adverse postoperative outcomes, these results support the role of PBM strategies focused on early identification and optimisation of anaemia as part of perioperative care.

### Study Limitations

Several constraints should be considered when interpreting these findings. First, the retrospective design limits causal inference, and certain potentially relevant confounders, including iron indices (e.g., ferritin and transferrin saturation), were unavailable. Additionally, the aetiology of anaemia (e.g., iron deficiency, chronic disease, or nutritional deficiency) could not be determined, precluding risk stratification by anaemia subtype.

Direct measurements of intraoperative blood loss were not consistently recorded, limiting our ability to compare blood loss between groups. In addition, the absence of a standardized institutional transfusion protocol and the retrospective nature of the study precluded precise determination of the relative contribution of individual clinical criteria to transfusion decisions. Alternative perioperative anaemia treatments, such as intravenous iron supplementation, were not routinely used during the study period, which may have contributed to the observed reliance on red blood cell transfusion.

Moreover, data on adjunct blood-conservation strategies, including perioperative use of antifibrinolytic agents such as tranexamic acid, were not systematically available and therefore could not be incorporated into the analysis. This limitation may have influenced both transfusion practices and clinical outcomes.

Finally, owing to the number and heterogeneity of recorded complications and limited statistical power, individual complication categories were not analysed separately. Clinically meaningful adverse events were, instead, evaluated according to CDC criteria (≥ grade II), with acute kidney injury and intensive care unit admission analysed as key outcomes.

Despite these limitations, this study provides real-life information on the effects of preoperative anemia and transfusion procedures in TKA.

## Conclusion

Preoperative anaemia is a significant determinant of transfusion requirements in patients undergoing TKA, and substantial physician-related variability exists in transfusion practices. These findings emphasise the importance of systematic preoperative anaemia screening, appropriate optimisation strategies, and the implementation of structured PBM programmes in orthopaedic surgery. Future multicentre prospective studies are required to confirm these observations and to establish standardised, evidence-based transfusion thresholds that improve the consistency of care and enhance perioperative patient safety.

## Ethics

**Ethics Committee Approval:** Ethical approval has been obtained from the Aksaray University Health Sciences Scientific Research Ethics Committee (approval no.: SAGATİK 2025-001, date: 30.01.2025).

**Informed Consent:** Preoperative, intraoperative, and postoperative data were retrospectively retrieved from the medical records of patients who had provided informed consent.

## Figures and Tables

**Table 1. Patient Demographic Data:** 

-	**Mean ± SD**	**Median (min-max)**
**Age (years)**	65.5±8.4	67 (45-89)
**BMI**	32.3±4.9	31.3 (22-47.8)
**Sex, n (%)** Female Male	237 (89.4) 28 (10.6)	-
**ASA physical status, n (%)** I II III	16 (6.0) 223 (84.2) 26 (9.8)	-
**Anaesthesia management, n (%)** Regional anaesthesia General anaesthesia	251 (94.7) 14 (5.2)	-
**Procedure type, n (%)** Unilateral Bilateral	154 (58.1) 111 (41.9)	-
**Procedure type, n (%)** Primary surgery Revision surgery	240 (90.6) 25 (9.4)	-
**Preoperative anaemia, n (%)**	114 (43.0)	-
Baseline Hb (g dL^-1^)	13.2 (9.1-18.3)	-
Intraoperative RBC units transfused	0.14±0.35	0 (0-1)
Postoperative RBC units transfused	1.15±1.5	1 (0-11)

ASA, American Society of Anaesthesiologists; BMI, body mass index; Hb, haemoglobin; RBC, red blood cell; SD, standard deviation; min-max, minimum-maximum

**Table 2. Distribution of Patients and Clinical Characteristics According to Preoperative Anaemia Status:** 

**Variables**	**Non-anaemic group** **(n = 151)**	**Anaemic group** **(n = 114)**	***P* value**
**Age (years), median (min-max)**	65 (48-84)	67.5 (45-89)	0.234^a^
**Female, n (%)^b^**	128 (84.8)	109 (95.6)	0.004^c^
**BMI, median (min-max)**	32.4 (22-47.8)	31.2 (23.4-45.4)	0.056^a^
**ASA physical status, n (%)^b^** 1 2 3	- 11 (7.3) 127 (84.1) 13 (8.6)	- 5 (4.4) 96 (84.2) 13 (11.4)	- 0.491^c^
**Laterality, n (%)^b^** Unilateral Bilateral	- 90 (59.6) 61 (40.4)	- 64 (56.1) 50 (43.9)	- 0.572^c^
**Surgical type, n (%)^b^** Primary surgery Revision surgery	- 140 (92.7) 12 (7.3)	- 100 (87.7) 14 (12.3)	- 0.168^c^
Duration of surgery (minute), median (min-max )	75 (30-150)	75 (12-140)	0.552^a^
**Comorbidities, n (%)^b^** Hypertension Diabetes mellitus Pulmonary disease Coronary artery disease Chronic kidney disease Neurological disorder	73 (48.3) 56 (37.1) 25 (16.6) 28 (18.5) 4 (2.6) 3 (2.0)	72 (63.2) 56 (49.1) 24 (21.1) 31 (27.2) 4 (3.5) 6 (5.3)	- 0.016^c^ 0.050^c^ 0.351^c^ 0.094^c^ 0.729^d^ 0.179^d^

^a^: Mann-Whitney U test; ^b^: column percentage; ^c^: Pearson’s chi-square test; ^d^: Fisher’s exact test; ASA, American Society of Anaesthesiologists; BMI, body mass index; min-max, minimum-maximum

**Table 3. Haemoglobin Values and Changes According to Anaemia Status:** 

**Haemoglobin parameters (g dL^-1^)**	**Non-anaemic group (n = 151)**	**Anaemic group (n = 114)**	***P* value**
Preop Hb	13.8 (13.0-18.3)	12.2 (9.1-12.9)	<0.001^a^
Postop Hb	12.2±1.2	10.5±1.2	<0.001^b^
Nadir Hb	9.9 (5.7-13.6)	9.1 (6.7-12.5)	<0.001^a^
Discharge Hb	10.6±1.2	10.2±0.9	0.002^b^

^a^: Mann-Whitney U test; ^b^: Student t-test; Hb, haemoglobin

**Table 4. Relationship Between Preoperative Anaemia and Blood Transfusion:** 

-	**Non-anaemic group** **(n = 151)**	**Anaemic group** **(n = 114)**	***P* value**
**At least one transfusion, n (%)**	82 (54.3)	79 (69.3)	0.013^a^
**Among patients who received transfusions, n (%)** Intraoperative only Postoperative only Intraoperative and postoperative	- 11 (13.4) 62 (75.6) 9 (11.0)	- 7 (8.9) 62 (78.5) 10 (12.7)	0.642^a^

^a^: Pearson chi-square test

**Table 5. Multivariable Logistic Regression Analysis for Factors Associated with Perioperative Blood Transfusion:** 

**Variable**	**OR**	**95% CI**	***P* value**
Age (per year increase)	**1.06**	1.02-1.10	**0.002**
Sex (female vs male)	0.53	0.21-1.32	0.171
Surgical type (bilateral vs unilateral)	**5.98**	3.13-11.43	**<0.001**
Revision surgery	2.19	0.81-5.91	0.122
Hypertension	0.61	0.33-1.15	0.127
Diabetes mellitus	1.05	0.59-1.87	0.862
Pulmonary disease	0.66	0.32-1.35	0.256
Coronary artery disease	**2.61**	1.18-5.77	**0.018**
Chronic kidney disease	8.88	0.79-99.64	0.077
Neurological disease	0.39	0.08-1.82	0.229
Anaesthesia type (regional vs general)	0.56	0.14-2.25	0.412

Model performance: Nagelkerke R² = 0.24; Cox & Snell R² = 0.18; Hosmer-Lemeshow goodness-of-fit test,* P*=0.843

ORs with 95% CIs were calculated using multivariable logistic regression. A *P* value <0.05 was considered statistically significant

CI, confidence interval; OR, odds ratio

**Table 6. Diagnostic performance of preoperative haemoglobin levels for predicting perioperative transfusion:** 

**Variable**	**Cut-off**	**Sensitivity**	**Specificity**	**PPV**	**NPV**	**Accuracy**	**AUC**	**95% CI**	***P* value**
Hb (g dL^-1^)	13.15	55%	64%	70%	48%	58 %	0.61	0.54-0.68	0.002

AUC, area under the curve; CI, confidence interval; Hb, haemoglobin; NPV, negative predictive value; PPV, positive predictive value
